# A Rare Presentation of Invasive Nontypeable *Haemophilus influenzae* With Systemic Involvement: A Case Report

**DOI:** 10.1155/crdi/1817056

**Published:** 2025-07-13

**Authors:** Hajra Nosheen, Lochan Bellamkonda, John George Youssef, Abhijeet Ghatol, Shagufta Naz Ali

**Affiliations:** ^1^Internal Medicine, McLaren Flint Hospital, Flint, Michigan, USA; ^2^Pulmonary and Critical Care, McLaren Flint Hospital, Flint, Michigan, USA; ^3^Infectious Disease, McLaren Flint Hospital, Flint, Michigan, USA

**Keywords:** *H. influenzae bacteremia*, *H. influenzae polyarthritis*, nontypeable *H. influenzae*

## Abstract

*Haemophilus influenzae* (*H. influenzae*) is a common organism that causes noninvasive infections in the respiratory tract. Lately, there has been an increasing incidence of invasive diseases with nontypeable strains not covered by Hib vaccines. We discuss a case of a middle-aged male with sickle cell trait and gout who presented with altered mentation and polyarthralgia. On investigations, he had *H. influenzae* bacteremia on two sets of blood cultures with dissemination to meninges and joints, causing acute encephalopathy and polyarthralgia. The initial results of arthrocentesis and lumbar puncture were sterile. However, PCR analysis of the fluid revealed nontypeable *H. influenzae.* The patient required mechanical ventilation and vasopressor support given sepsis but recovered after extended antibiotics and multiple surgical debridements. Our case highlights the importance of maintaining a high suspicion of invasive disease in decompensated patients and the role of advanced diagnostics in treatment and outcome.

## 1. Introduction

*Haemophilus influenzae* is a common upper respiratory pathogen that most frequently presents with otitis media, bronchitis, or pneumonia [1]. Most cases of invasive *H. influenzae* were previously associated with type b. Since the implementation of childhood *Haemophilus influenzae* type b (Hib) vaccination, the infection rates have dropped significantly, and nontypeable *H. influenzae* has become an increasingly common cause of invasive disease [1]. The vaccine does not help prevent infection caused by nontypeable variants, which have been increasingly reported to cause meningitis [2, 3] and arthritis [4–11]. To our knowledge, there are no reported cases of invasive nontypeable *H. influenzae* resulting in septic arthritis, bacteremia, and meningitis.

## 2. Case Description

We present a case of an African-American male in his late fifties with a past medical history of hypertension, diabetes, hyperlipidemia, gout, and sickle cell trait who presented to our facility with worsening polyarthralgia involving the bilateral knees, ankles, and wrists for 3 days and altered mentation for one day. He had previously been treated as an outpatient with oral steroids and NSAIDs without improvement in his joint pains.

Upon initial evaluation, he was hypotensive and tachycardic with intermittent low-grade fevers (37.6°C–38°C), which raised concerns for an infectious etiology. Initial infectious evaluation with urinalysis and chest imaging was unremarkable. He received fluid resuscitation consistent with current sepsis guidelines, and two sets of blood cultures were collected. Despite appropriate fluid resuscitation, the patient continued to remain hypotensive and required vasopressor support with norepinephrine and vasopressin. The primary concern was the infectious process, and he was empirically started on a combination of vancomycin and piperacillin/tazobactam therapy.

Several hours after the presentation, the patient's mentation worsened, and he required intubation followed by mechanical ventilation. On neurological examination, he was unresponsive. His pupils were round and symmetric, with positive oculocephalic reflex and corneal reflex. No signs of meningeal irritation were appreciated. Due to his declining mentation and concomitant multiorgan failure, there was a concern for central nervous system (CNS) involvement, and piperacillin/tazobactam was discontinued in support of cefepime and doxycycline to cover atypical pathogens. Within 12 h of admission, the patient was noted to be oliguric with worsening renal function and was subsequently started on continuous renal replacement therapy.

By the following day, the two sets of blood cultures grew beta-lactamase-negative *H. influenzae*. Due to the patient's worsening mentation and polyarthralgia at presentation, a lumbar puncture was performed on the third day of admission and antibiotics. The cerebrospinal fluid (CSF) was clear, and the analysis revealed glucose levels of 106 mg/dL (40–70 mg/dL), protein levels of 54 mg/dL (15–45 mg/dL), and nucleated cell counts of 3/μL (0–5/μL). Arthrocentesis of the knee joint revealed turbid synovial fluid with total nucleated cells of 459,232/UL (0–200/UL), polymorphonuclear leukocyte count of 98%, and no crystals. There was no growth on a culture medium for either sample. As the patient developed thick secretions with mucous plugging of the airways, bronchoscopic evaluation with bronchoalveolar lavage was also performed. However, bronchoscopic cultures were unremarkable (Table 1).

Despite aggressive support, the patient continued to decline with worsening hypotension, which necessitated the addition of continuous epinephrine infusion. Due to the persistent concern for infection, a polymerase chain reaction (PCR) test was conducted on the CSF and synovial fluid. Moreover, his blood cultures were sent to the Department of Health and Human Services (DHHS) for serotyping of *H. influenzae.* While awaiting the results of PCR testing, the patient was subsequently taken to the operating room for surgical debridement of the bilateral knees and right ankle, as well as aspiration of the bilateral wrists.

Following surgical intervention, the patient demonstrated rapid clinical improvement and was able to tolerate removal from vasopressors and mechanical ventilation within 48 h. After 12 days of cefepime and doxycycline, the antibiotics were switched to ceftriaxone and subsequently de-escalated to ampicillin-sulbactam to complete a 6-week course. He also transitioned to intermittent hemodialysis as his renal function improved. Several days later, PCR testing showed the presence of *H. influenzae* in both the CSF and the synovial fluid. His serotyping from DHHS resulted in nontypeable *H. influenzae.* He was discharged to a rehabilitation facility approximately 16 days after his initial presentation and 1 week after surgical debridement to complete his antibiotics.

At his follow-up visit, the patient has been recovering well. He has demonstrated improved mobility of bilateral joints and is actively engaged in physical therapy. Unfortunately, his kidney function did not recover, and he now requires hemodialysis 3 days per week.

## 3. Discussion

*H. influenzae* is a Gram-negative coccobacillus, divided into encapsulated (typeable a-f) and unencapsulated (nontypeable) strains. The unencapsulated type has the potential to cause invasive disease. An increasing number of invasive diseases have been reported worldwide, predominantly in young and older populations, with possible mechanisms of vaccine-mediated strain replacement, improved bacterial detection, increased virulence, and demographic changes [12].

Nontypeable *H. influenzae* frequently causes pneumonia, followed by bacteremia and meningitis in middle-aged and elderly patients with higher case fatality [13]. Polyarticular septic arthritis is a rare presentation of nontypeable *H. influenzae.* There have been reports of septic arthritis associated with nontypeable *H. influenzae* in patients with risk factors [4, 5, 7–11]. Known risk factors for invasive disease include pregnancy, alcoholism, trauma, rheumatologic disease, malignancy, and immunodeficiency. Reports of invasive nontypeable *H. influenzae* infection in patients without risk factors have been reported [6] but are much less common (Table 2).

Our patient did report a history of sickle cell trait; however, imaging did not reveal any splenic infarction. He had a normal reticulocyte count and no schistocytes on the peripheral smear. He reported a history of vaccination against Hib. Another predisposing factor was gout, which is well-documented to be associated with risk for septic arthritis [14]. Moreover, he was immunocompetent with unremarkable immunoglobulins, fungal panel, antinuclear antibody, and HIV testing.

As we found in our case, septic arthritis creates a complex clinical picture, particularly in those with underlying rheumatologic disease [14]. The patient in our case was initially treated outpatient with NSAIDs and steroids due to suspicion of a gout flare before presenting to the hospital with worsening symptoms that resulted in the delay of appropriate treatment. *H. influenzae* is also reported to be associated with reactive arthritis, which further complicates diagnosis [15, 16]. As noted in our case and others, cultures may appear falsely sterile, leading to incorrect diagnoses and management without a high level of suspicion of infection.

Per our literature search, this is the first case of nontypeable *H. influenzae* in which dissemination to blood, meninges, and multiple joints was noted. This report highlights concerns regarding the rise of invasive diseases caused by unencapsulated strains, emphasizing the need for a vaccine for high-risk populations.

## 4. Conclusion

Our case demonstrates a rare presentation of invasive *H. influenza*e septic arthritis with dissemination to the blood and CNS. Although the culture evaluation of spinal fluid and synovial fluid in our case appeared sterile, the initial bacterial growth in blood cultures maintained a high level of suspicion for infection and prompted further testing with the PCR. In patients with systemic symptoms, evaluating any potential source of infection with PCR testing is necessary if the suspicion remains high despite initial cultures appearing sterile.

## Figures and Tables

**Table 1:** Trend of daily labs from the day of admission to day 15th.

Labs with reference range	Day 1	Day 5	Day 10	Day 15
White blood cells (4.50–10.0 × 10^3^/μL)	3.67 × 10^3^/uL	28.82 × 10^3^/uL	29.05 × 10^3^/uL	13.52 × 10^3^/uL
Hemoglobin (12.0–15.0 g/dL)	14.2 g/dL	18.8 g/dL	6.7 g/dL	8.0 g/dL
Platelets (140–440 × 10^3^/μL)	51 × 10^3^/uL	38 × 10^3^/uL	194 × 10^3^/uL	317 × 10^3^/uL
Sodium (135–145 mmol/L)	133 mmol/L	134 mmol/L	139 mmol/L	135 mmol/L
Potassium (3.5–5.5 mmol/L)	4.6 mmol/L	4.7 mmol/L	4.7 mmol/L	5.3 mmol/L
Bicarbonate (21.6–31.8 mmol/L)	16.9 mmol/L	24 mmol/L	22 mmol/L	20.9 mmol/L
Blood urea nitrogen (9.0–27.0 mg/dL)	117 mg/dL	40.0 mg/dL	21 mg/dL	86.6 mg/dL
Creatinine (0.6–1.5 mg/dL)	6.0 mg/dL	2.1 mg/dL	2.2 mg/dL	3.6 mg/dL
ALT (10–49 U/L)	227 U/L	347 U/L	11 U/L	82 U/L
AST (14–35 U/L)	222 U/L	165 U/L	266 U/L	52 U/L
Lactate (0.5–2.0 mm/L)	5.5 mmol/L	2.1 mmol/L	1.3 mmol/L	—
CRP (0.00–0.80 mg/dL)	54.00 mg/dL	21.40 mg/dL	—	6.20 mg/dL
ESR (0–20 mm/h)	> 130 mm/h			66 mm/hr
Procalcitonin (0.02–0.50 ng/mL)	> 100.00 ng/mL			2.67 ng/mL

*Note:* ALT: alanine aminotransferase, AST: aspartate aminotransferase.

Abbreviations: CRP, C-reactive protein; ESR, erythrocyte sedimentation rate.

**Table 2:** Summary of previously reported cases of nontypeable *H. influenzae* polyarticular septic arthritis.

Case reports	Year	Age/sex	Risk factors	Joint involvement	Definitive treatment
[11]	1979	46/F	Multiple myeloma	Bilateral knees, right ankle, right elbow, and left wrist	Arthrocentesis + ampicillin
[10]	1991	40/M	Alcohol use	Bilateral knees, bilateral ankles, left elbow, left wrist, and left knee	Arthrocentesis + cefuroxime
[9]	1991	35/M	Common variable immunodeficiency	Left knee	Arthrocentesis + aztreonam
[8]	1998	46/F	Rheumatoid arthritis, steroids	Right shoulder, right elbow, right knee, left wrist, and left hand	Arthrocentesis + ciprofloxacin
[7]	2006	57/M	Gout, laryngeal cancer, alcohol use	Right knee and left wrist	Arthrocentesis + levofloxacin, and ampicillin
[6]	2011	18/M	None	Bilateral elbows, knees, wrists, and hips	Surgical debridement + ampicillin
[5]	2019	56/M	Gout	Bilateral wrists, ankles, and right knee	Surgical debridement + ceftriaxone
[4]	2021	55/M	HIV	Bilateral knees, left wrist, and left ankle	Surgical debridement + ceftriaxone
Our case	2024	Middle age/M	Sickle cell trait, gout	Bilateral ankles, knees, and wrists	Surgical debridement + ceftriaxone and ampicillin-sulbactam

## Data Availability

The data that support the findings of this study are available on request from the corresponding author. The data are not publicly available due to privacy or ethical restrictions.
